# Evaluation Physical Characteristics and Comparison Antimicrobial and Anti-Inflammation Potentials of Dental Root Canal Sealers Containing Hinokitiol *In Vitro*

**DOI:** 10.1371/journal.pone.0094941

**Published:** 2014-06-10

**Authors:** Yin-Hua Shih, Dan-Jae Lin, Kuo-Wei Chang, Shih-Min Hsia, Shun-Yao Ko, Shyh-Yuan Lee, Shui-Sang Hsue, Tong-Hong Wang, Yi-Ling Chen, Tzong-Ming Shieh

**Affiliations:** 1 Institute of Oral Biology, School of Dentistry, National Yang-Ming University, Taipei, Taiwan; 2 Department of Dental Hygiene, College of Health Care, China Medical University, Taichung, Taiwan; 3 Department of Dentistry, School of Dentistry, National Yang-Ming University, Taipei, Taiwan; 4 Department of Stomatology, Oral and Maxillofacial Surgery, Taipei Veterans General Hospital, Taipei, Taiwan; 5 School of Nutrition and Health Sciences, Taipei Medical University, Taipei, Taiwan; 6 Graduate Institute of Medical Science, College of Health Science, Chang Jung Christian University, Tainan, Taiwan; 7 Department of Oral Pathology, China Medical University Hospital, Taichung, Taiwan; 8 Tissue Bank, Chang Gung Memorial Hospital, Tao-Yuan, Taiwan; Monash University, Australia

## Abstract

Hinokitiol displays potent antimicrobial activity. It has been used in toothpaste and oral-care gel to improve the oral lichen planus and reduce halitosis. The aim of this study was to evaluate the antimicrobial activity of 3 different dental root canal sealers with hinokitiol (sealers+H) and their physical and biological effects. AH Plus (epoxy amine resin-based, AH), Apexit Plus (calcium-hydroxide-based, AP), and Canals (zinc-oxide-eugenol-based, CA), were used in this study. The original AH and CA exhibited strong anti-methicillin-resistant *Staphylococcus aureus* (anti-MRSA) activity, but AP did not. The setting time, working time, flowability, film thickness, and solubility of each sealer+0.2%H complied with ISO 6876:2001. CA+0.2%H exhibited high cytotoxicity, but the others sealers+0.2%H did not. Because hinokitiol combined with Zn^2+^ in CA creates a synergistic effect. The physical tests of AP+0.5%–1%H complied with ISO 6876:2001, improved antimicrobial activity, inhibited inflammation genes *cyclooxygenase-2* (COX-2) and *hypoxia-inducible factor-1α* (HIF-1α) mRNA in MG-63 cells and human gingival fibroblasts (HGF), and down-regulated *lysyl oxidase* (LOX) mRNA of HGF. In summary, AH and CA demonstrated strong antimicrobial activity, but AP did not. Application of hinokitiol increases AH anti-MRSA activity should less than 0.2% for keep well flowability. AP+0.5%–1% hinokitiol exhibited strong physical, antibacterial, and anti-inflammation potentials, and inhibited *S. aureus* abscess formation. Applying an appreciable proportion of hinokitiol to epoxy-amine-resin-based and calcium-hydroxide-based root canal sealers is warranted, but the enhanced cytotoxicity and synergistic effect must be considered.

## Introduction

Most endodontic therapy failures occur because of microleakage of irritants from diseased root canals [1]. Microbes are the main etiologic factors of pulpitis; therefore, microorganisms must be removed from the root canal. Consequently, the use of root canal filling materials with antibacterial activity is considered beneficial to reduce the number of remaining microorganisms and to eradicate infection. Five types of dental root canal sealer are used in clinical settings: resin-based, calcium-hydroxide-based, zinc-oxide-eugenol-based (ZnO-eugenol), glass-ionomer-based, and silicone-based sealers. Studies have been conducted to assess the antimicrobial activity of various endodontic sealers [2], [3], but few studies exist on the antimicrobial properties of sealers mixed with natural antibacterial compounds.

Hinokitiol is a natural component isolated from *Chamacyparis taiwanensis.* Hinokitiol exhibits antibacterial, antifungal, antiviral, and insecticidal activities [4]–[6] and no developmental toxicity or carcinogenic effects have been observed [7], [8]. Hinokitiol inhibits oral bacteria but exhibits low cytotoxicity to normal oral cells [9], [10], and has been used in toothpaste and oral-care gel to improve the oral lichen planus and reduce halitosis [11]–[13]. Hypoxia-inducible factor-1α (HIF-1α), prostaglandins, and tumor necrosis factor-α (TNF-α) mediated inflammatory response have also been inhibited by hinokitiol [14]–[16].

Inflammation and high vascular density contribute to tissue edema and result in an overall increase in tissue volume. Approximately twenty percent gram-negative microbes live in infected root canals. The lipopolysaccharides (LPS) produced by gram-negative bacteria induce HIF-1α mRNA expression [17]. Positive expression of HIF-1α has been observed in inflammatory periodontal pockets [18], [19]. HIF-1α is a member of the HIF family, HIF-1 increases the transcription of several genes for proteins that promote blood flow and inflammation, including vascular endothelial growth factor (VEGF), heme oxygenase-1, endothelial and inducible nitric oxide synthase (NOS), cyclooxygenase-2 (COX-2), and lysyl oxidase (LOX) [20]. COX-1 and COX-2 are the key enzymes in prostaglandins biosynthesis in mediator-implicated inflammation. The activation of COX-2 expression might contribute to the pathogenesis of root canal sealer induced periapical inflammation [21]. Interleukin-1β (IL-1β) up-regulates HIF-1α and has been observed to be up-regulated by nuclear factor κB (NFκB) and the COX-2 inflammatory signaling pathway [22], [23]. LOX initiates the cross linking of collagen and elastin, and is up-regulated by HIF-1. LOX is associated with a critical and formerly unrecognized role in *S. aureus* abscess formation [24]. HIF-1α, COX-2, and LOX expression is related to inflammation [23]–[25].

Root canal sealers must have the correct physical, biological, and antimicrobial properties. The physical characteristics of sealers should comply with ISO 6876:2001 (endodontic filling material) to facilitate ease of use and clinical indication. The biological effects of sealers should not be harmful to the periradicular tissues of patients. Antimicrobial properties can ameliorate bacterial infection and reduce inflammation. These properties influence the quality of the root canal filling. Enhanced sterilization and inhibited inflammation increase endodontic therapy success rates. The purpose of this study was to compare the antimicrobial activity of resin-based, calcium-hydroxide-based, and ZnO-eugenol-based sealers, and determine the optimal hinokitiol/sealer ratio. The optimal ratio was evaluated according to physical, cytotoxicity, antimicrobial, and inflammation gene expression tests.

## Materials and Methods

### Microorganisms Culture & Bacterial Growth Curve

Methicillin-resistant *Staphylococcus aureus* (MRSA, ATCC number: 33591), *Aggregatibacter actinomycetemcomitans* (ATCC number: 33384), and *Streptococcus mutans* (ATCC number: 25175) were used in the study. The MRSA and *S. mutans* were cultured in tryptic soy broth (TSB). The *A. actinomycetemcomitans* was cultured in brain heart infusion (BHI) broth. The culture methods and bacterial growth curve analysis using the kinetic microplate method were the same as in the previous study [9].

### Dental Sealers and Specimens Preparation

Three root canal sealers, AH Plus (epoxy-amine resin-based, AH), Apexit Plus (calcium hydroxide-based, AP), and Canals (ZnO eugenol-based, Grossman's sealer, CA) were used in the study. The components of the sealers are list in Table 1. Each sealer specimen prepared complied with the commercial operation manual. The 3 sealers with the various weight ratios of hinokitiol (0.2%H, 0.5%H, 1%H, and 2%H) are presented in Table S1.

**Table 1:** Commercial dental sealer components list.

Products	Indications	Components	Manufacturer
AH	Root canal sealing material (epoxy-amine resin based)	Paste A (epoxy): diglycidil-bisphenol-A-ether, calcium tungsten, zirconium oxide, aerosol, iron oxide; Paste B (amina): amina 1-adamantane, N, N-Dibenzyl-5-oxanonandiamine-l,9, TCD-diamine, calcium tungsten, zirconium oxide, silicone oxide	DeTrey/Dentsply, Konstanz, Germany
AP	Root canal sealer (calcium hydroxide based)	Mixed paste (approx.): Ca(OH)_2_ 15.9%, hydrogenated colophony 15.8%, silicon dioxide 0.4%, salicylate resins, ethyl toluene sulphonamide	Ivoclar Vivadent, Schaan, Liechtenstein
CA (Grossman's sealer)	Root canal filling material (zinc oxide–eugenol based)	Powder: zinc oxide, bismuth subcarbonate; Liquid: clove oil, peanut oil	Showa Yakuhin Kako Co, Japan

### Setting Time

Filled 200 µL of the sealers into a stainless steel ring mold incorporating a cavity (d = 10 mm, h = 2 mm) and waited for 2 min. The specimens were maintained at 37°C and a relative humidity of not less than 95%. The specimens were tested for hardness with a 100 g indenter (the flat end of diameter was 2 mm, and the cylindrical tip was 5 mm), which was placed vertically on the horizontal surface of the sealer. The indenter tip was cleaned and the same operation was repeated until no indentations occurred. The setting time was defined as the time from the end of the mixing process until the time no indentations were made on the specimen by the indenter.

### Working Time

The sealing materials were set in normal atmosphere over several time frames to analyze their flowability. After 10 min, the weight was removed and the diameters of the compressed specimens were measured. Working time was defined as the time taken for the specimens to shrink by 10%.

### Flowability

Injected 50 µL of the well-mixed sealing materials in the center of one of the glass plates (40 mm×40 mm, h = 5 mm). The material was left to set in a normal atmosphere for 3 min, covered with another glass plate, and weighted with a 100 g weight. After 10 min, the weight was removed, and the maximum and minimum diameters of the compressed specimens of the sealers were measured. The difference between the maximum and minimum diameters should be less than 1 mm. The mean of the 2 diameters were recorded. According to ISO 6876:2001, the diameter needs not to be less than 20 mm.

### Film Thickness

The measurement of the combined thickness of the 2 glass plates (200 mm^2^, h = 5 mm) required an accuracy of 1 µm. A volume of 20 µL of the sealer was deposited on the center of the glass plate and immediately covered with another glass plate. Three minutes from the start of the mixing process, a 10 kg weight was placed vertically on the glass plate to ensure the gap between the 2 plates was completely filled with the sealer. Ten minutes from the start of the mixing process, the thickness of the 2 glass plates (N_0_), and the 2 glass plates and the film of sealer (N_1_) were measured using the micrometer. The film thickness was N_1_–N_0_. The film thickness needed to be less than 50 µm to comply with ISO 6876:2001.

### Solubility

The specimens were prepared using the setting time method. Two minutes from the completion of the mixing process, the specimens were maintained at 37°C and a relative humidity of not less than 95% for 24 h. After recording the weight of each specimen, they were immersed in 50 mL of water in a 9-cm petri dish, and maintained at 37°C and a relative humidity of not less than 95% for 24 h. The water was evaporated at a temperature of 65°C overnight, and the specimens were dried at 110°C until the weight was stable. The final specimen weight was then recorded. The weight loss the specimen incurred in the water may not be more than 3% of the maximum weight, according to ISO 6876:2001.

### Cell Culture

The human gingival fibroblasts (HGF) were obtained from discarded human gingival connective tissues and approved by an institutional review board (China Medical University Hospital, DMR98IRB-158). The HGF cells culture was followed previous study [26]. Human osteosarcoma cell lines MG-63 (BCRC NO. 60279) and HGF were cultured in Dulbecco's modified eagle medium (DMEM) containing 10% heat-inactivated fetal calf serum (FCS), 100 U/mL penicillin G sodium, 100 µg/mL streptomycin sulphate, and 0.25 µg/mL amphotericin B at 37°C in a humidified incubator containing 5% CO_2_. The cells were washed with phosphate buffered saline (PBS), then trypsinized and subcultured when near confluence.

### Cell Viability Assay

The specimens were prepared using the setting time method and wait for setting of each dental sealer. Extraction of three prepared specimens of each based dental sealer were immersed after setting in 10 mL cell culture medium by using the ratio of 0.7 cm^2^/mL at 37°C for 3 d. The fresh conditional medium was collected for the 3-(4,5-dimethylthiazol-2-yl)-2,5-diphenyltetrazolium bromide (MTT) assay [27]. MG-63 10^4^ cells/100 µL were inoculated and cultured for 20–24 h at 37°C in 96-well tissue culture plates. The cells were treated with 2 folds serial dilution of these extraction media (0%, 25%, 50%, and 100%) for 24 h, 3-days, and 7-days for sealer cytotoxicity analysis. The cells were treated/co-treated with hinokitiol (6.25–400 µM), CaCl_2_ (6.25–400 µM), ZnO (6.25–400 µM), hinokitiol+CaCl_2_, and hinokitiol+ZnO for 24 h for drug synergism test. After removing the culture medium, MTT assay were performed according to previously used protocols [28].

### Agar Diffusion Test

Liquid 1.5% agar broth was equilibrated in a 50°C water bath for 30 min following autoclave sterilization. Cultures were inoculated with 10^6^ CFU/mL of glycerol stock by swirling, before being poured into the plates. A volume of 20 µL un-setting dental sealer specimens was placed on the surface of the solidified TSB agar, and the cultures were incubated for 24 h at 37°C. The diameter of the inhibition zone was recorded and photographed [9].

### MRSA and Sealers Direct Contact Test

The effect of close contact between test bacteria and the sealers on the kinetics of bacterial outgrowth was analyzed by the direct contact test [29], [30]. A 96 well plate was held vertically, and coated with 20 µL freshly mixed test materials on the side wall of the wells (Group A wells). The samples were allowed to set for each setting time before testing. Ten µL of the bacterial suspension (10^6^ CFU/mL) direct contact on the samples, and the plate was incubated in a vertical position for 1 h at 37°C. Then, 245 µL TSB was added to each Group A well, and gently mixed for 2 min; 15 µL was then transferred from the Group A wells to an adjacent set of wells containing 215 µL fresh medium, designated as Group B. The final volume of both groups was 230 µL. Unused wells were filled with 230 µL of distilled water to inhibit broth evaporation. An 18 h kinetic analysis of the culture growth was performed at 37°C. The kinetic analysis included a 5-s shaking step before each of the time point measurements of OD600, at 30 min intervals, and analyzed using VersaMaxTM and Softmax Pro (version 5.4.1) software.

### Anti-inflammation Potentials Test

Half conditional medium (AP, AP+0.5%H, and AP+1%H) and half fresh cell culture medium were used as MG-63 culture for 24 h. RNA extraction, primer sequences, and reverse transcription-polymerase chain reaction (RT-PCR) were performed according to previously used protocols [21], [28], . The COX-2, HIF-1α, and LOX mRNA expression signals were normalized with glyceraldehyde 3-phosphate dehydrogenase (GAPDH). At least 3 independent PCR reactions were performed to validate the reproducibility of the analyses. Competitive PCR product signals were quantified using ImageQuant (v. 5.2, GE Healthcare Life Sciences).

### Statistical Analysis

The unpaired *t* test was used for analysis. Data were shown as the mean ± standard error. Differences between the variants were considered significant when *P*<0.05. CompuSyn software (Version 1.0, ComboSyn Inc., USA) was used to quantify synergism and antagonism in the drug combinations.

## Results

### Hinokitiol inhibits oral bacteria growth

**Figure 1:**
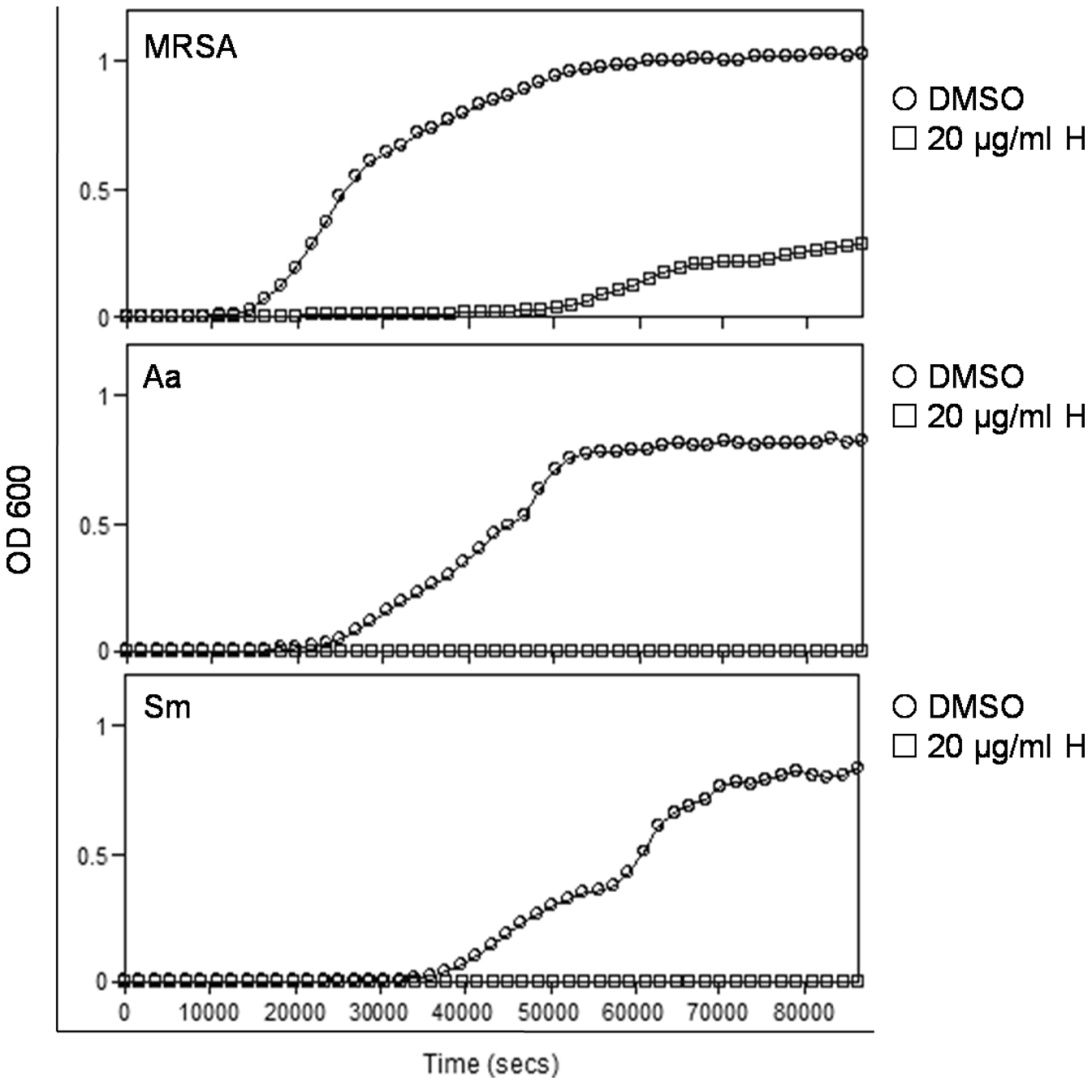
Hinokitiol inhibited oral bacterial growth. Bacterial growth curves after hinokitiol treatment for 24*A. actinomycetemcomitans* (Aa), and *S. mutans* (Sm). The y-axis is OD 600, and the x-axis is time (secs). Vehicle (0.1% DMSO) and 20 µg/mL hinokitiol (20 µg/mL H) were tested. Three independent experiments were performed in triplicate.

The result of the kinetic analysis showed that *A. actinomycetemcomitans* and *S. mutans* growth were completely inhibited, but MRSA was partially inhibited when treated with 20 µg/mL (approximately 120 µM) of hinokitiol (Fig. 1). MRSA exhibited a stronger tolerance to hinokitiol than *A. actinomycetemcomitans* and *S. mutans* did. Anti-MRSA activity was used to test the antibacterial activity of the sealers. According to this result, 100 fold hinokitiol (weight ratio 0.2% hinokitiol, 0.2%H) in the test sealers was used to test physical, biological, and antimicrobial effects.

### Physical characteristics of the sealers and sealers+0.2%H conformed to ISO 6876:2001

CA was the fastest-setting solid of all sealers tested in the study, followed by AP, and then by AH. Hinokitiol is a metal chelator. The setting times of AH, AP, and CA containing 0.2% hinokitiol (sealers+0.2%H) all increased slightly. The working times of the sealers and the sealers+0.2%H exhibited no substantial change. The flowability of all the sealers in the study was >20 mm in accordance with ISO 6876:2001, and decreased after 0.2% hinokitiol was mixed with each sealer. The flowability of AP+0.2%H and CA+0.2%H conformed to ISO 6876:2001, but that of AH+0.2%H did not. The maximum film thickness and solubility increased after 0.2% hinokitiol was mixed with each sealer, and they also conformed with ISO 6876:2001. The detailed physical characteristics of the sealer with and without 0.2% hinokitiol are listed in Table 2.

**Table 2:** Physical tests of the sealers contained 0.2% hinokitiol (Mean (SD)).

	Setting time (h)	Working time (h)	Flowability (mm)	Film thickness (µm)	Solubility (%)
ISO 6876:2001	-	-	>20 mm	<50 µm	<3%
AH	8.17 (0.29)	6.68 (0.48)	21.62 (1.85)	21.67 (5.03)	0.13 (0.06)
AH+0.2%H	8.33 (0.58)	6.70 (0.48)	18.05 (3.48)*	27.33 (1.53)	0.24 (0.03)*
AP	3.75 (0.50)	2.97 (0.15)	28.52 (1.42)	12.33 (0.58)	1.42 (0.32)
AP+0.2%H	4.00 (0.41)	2.70 (0.387)	25.13 (1.90)***	14.33 (2.08)	1.76 (0.72)
CA	2.00 (0.10)	1.50 (0.19)	26.07 (2.86)	10.67 (6.11)	1.32 (0.46)
CA+0.2%H	2.50 (0.10)	1.92 (0.32)	24.04 (1.62)	17.67 (7.37)	1.33 (0.51)

Entries are mean values with standard deviations in parentheses, and sample size (Mean (SD)). An unpaired *t* test was used to determine significant differences of the physical characteristics among the sealers contained various hinokitiol dosages in each group. The sample size of each group were 3 to 10. The asterisk indicates *P*<0.05, the double asterisk indicates *P*<0.01, and the triple asterisk indicates *P*<0.001.

### Cytotoxicity of the sealers+0.2%H increased

**Figure 2:**
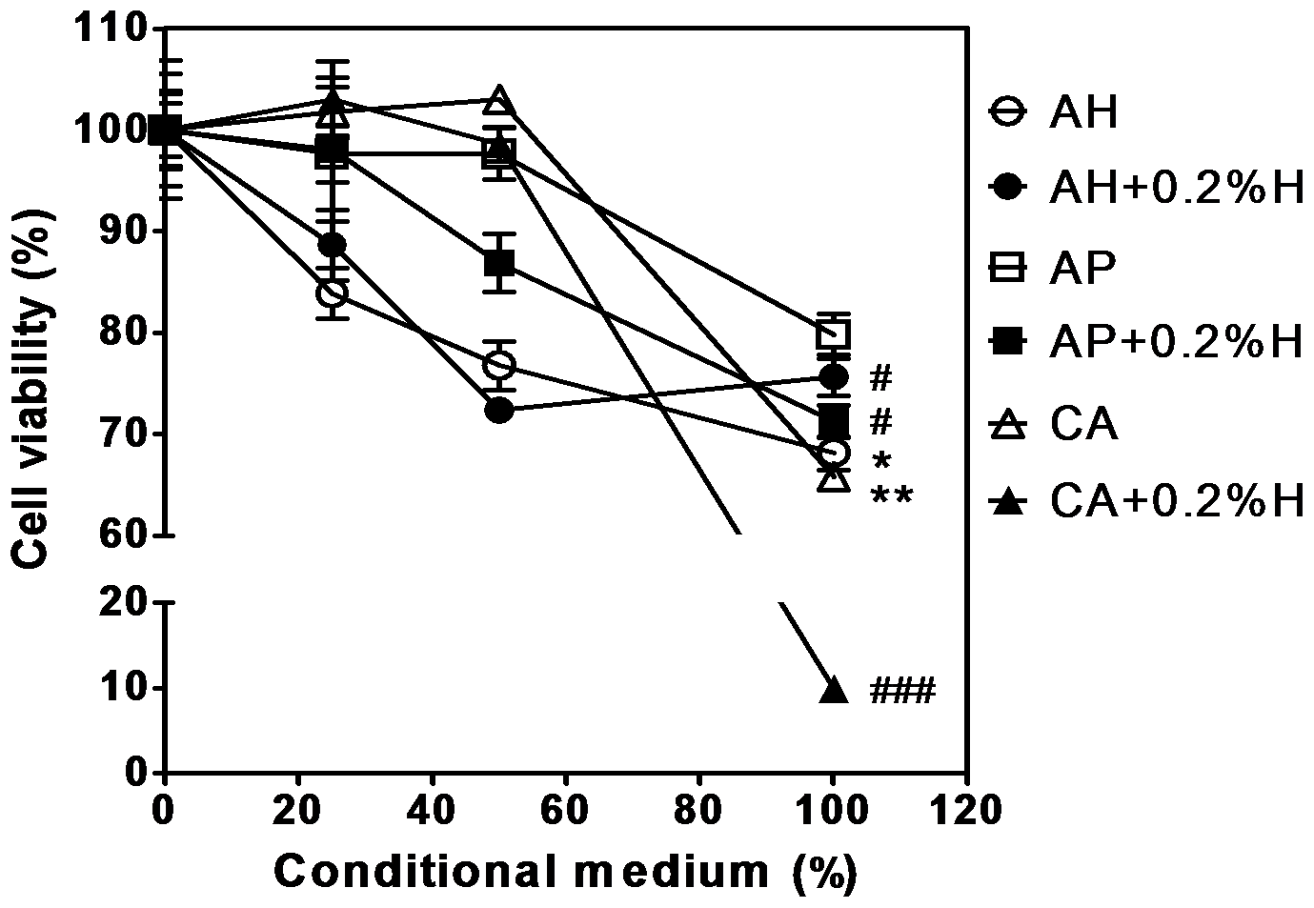
Cytotoxicity of dental canal sealers contain 0.2% hinokitiol were increased. The MG-63 were treated with the extract of the sealers (AH, AP, CA, AH+0.2%H, AP+0.2%H, and CA+0.2%H) conditional medium for 24 h, and the cytotoxicity was analyzed by MTT test. Two folds serial dilution of these conditional media (0%, 25%, 50%, and 100%) were tested. Three independent experiments were performed in triplicate. Unpaired *t* test. Significant difference between the different based sealers marked as “*”, and between the sealer and sealer+0.2%H marked as “#”. * or #, *P*<0.05; **, *P*<0.01; ###, *P*<0.001.

The MG-63 cell viability test after 100% conditional medium treatment revealed the following viability sequence: AP (79.83±3.516%) > AH (68.14±6.381%, *P*<0.05) > CA (65.76±1.245%, *P<*0.001). The conditional medium of the sealers+0.2%H, MG-63 cell viability of AH+0.2%H (75.65±3.203%, *P<*0.05) and AP+0.2%H (71.25±2.286%, *P<*0.05) decreased slightly, but that of CA+0.2%H decreased substantially (9.930±1.303%, *P*<0.001) (Fig. 2). After 50% conditional medium treatment, the MG-63 viabilities all decreased. The 50% conditional medium of CA and CA+0.2%H treatment was not cytotoxic to MG-63.

### Antibacterial activity of the sealers+0.2%H increased

**Figure 3:**
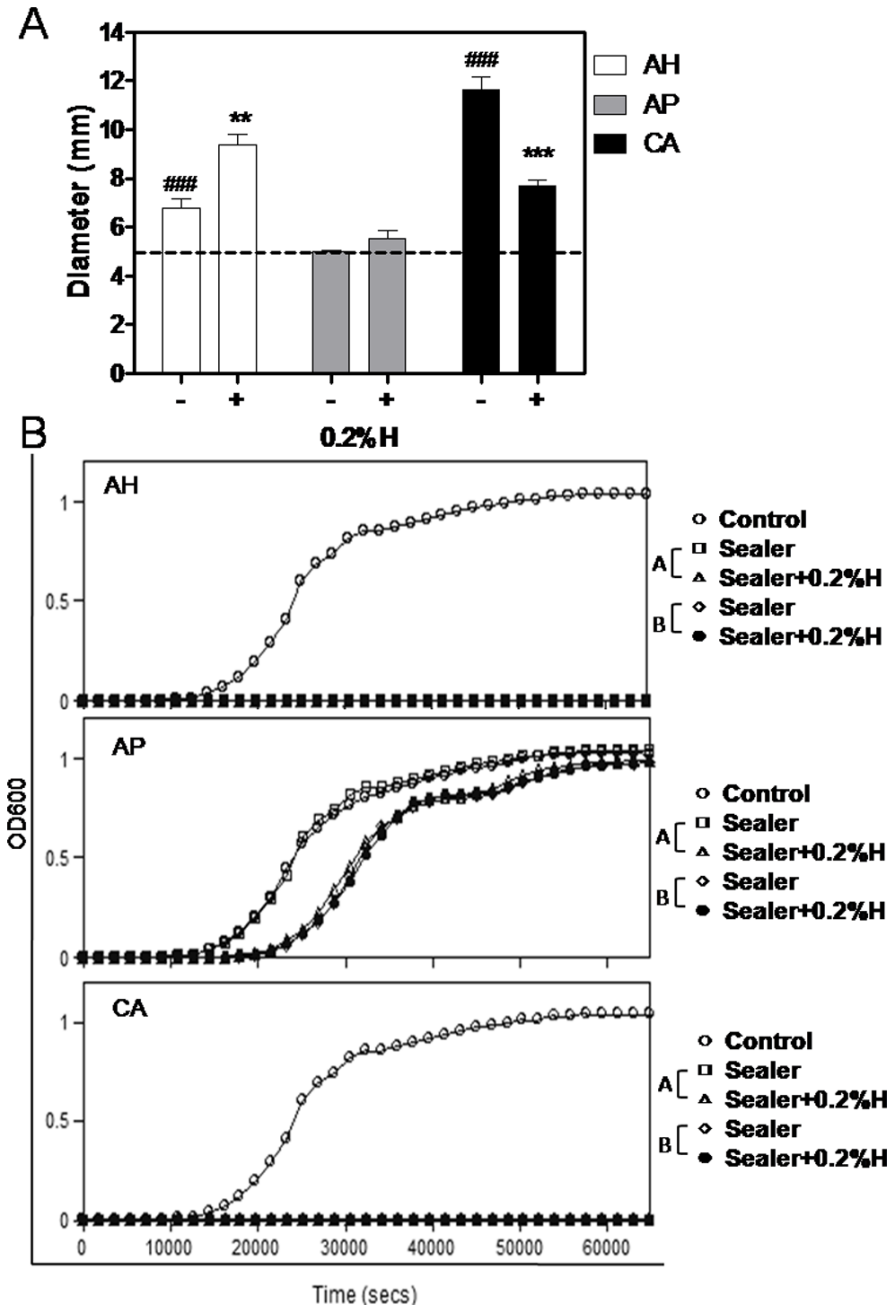
Anti-MRSA activity of AH Plus and Apexit Plus contain 0.2% hinokitiol were increased, but Canals contain 0.2% hinokitiol was not. (A) Agar diffusion test analysis of anti-MRSA activity of the sealers (AH, AP, CA, AH+0.2%H, AP+0.2%H, and CA+0.2%H). Dot line, the diameter of the specimen (5 mm); column, mean of more than triplicate analysis; bars, SE. (B) Direct contact test analysis of the effect of close contact between test bacteria and the sealers on the kinetics of bacterial outgrowth. Control was normal MRSA growth, A was Group A (with sealers in the wells), and B was Group B (transfer 15 µl TSB from Group A and without sealers in the wells). Unpaired *t* test. Significant difference between the different based sealers marked as “*”, and between the sealer and sealer+0.2%H marked as “#”. **, *P*<0.01; *** or ###, *P*<0.001.

An inhibition zone was clearly visible in AH (6.775±0.727 mm) and CA (11.650±0.981 mm), but not in AP (5.000±0.089 mm). Comparing the sealers and the sealers+0.2%H before setting, the inhibition zone diameters of AH+0.2%H increased (9.375±0.810 mm, *P*<0.01), and those of CA+0.2%H decreased (7.700±0.400 mm, *P*<0.001). No significant difference was recorded for AP+0.2%H (5.500±0.837 mm, *P*>0.05) (Fig. 3A). The bacterial outgrowth was monitored in both the presence (Group A) and absence (Group B) of the tested sealers, using the direct contact test. Both groups of AH and CA, with and without 0.2% hinokitiol, completely inhibited MRSA growth, but AP did not. MRSA was eliminated as a result of direct contact with AH+0.2%H and CA+0.2%H for 1 h. However, AP+0.2%H exhibited a minor increase in anti-MRSA activity in Groups A. The lag phase was extended in the AP+0.2%H group (Fig. 3B).

### Zn^2+^ and hinokitiol synergism of cytotoxicity

**Figure 4:**
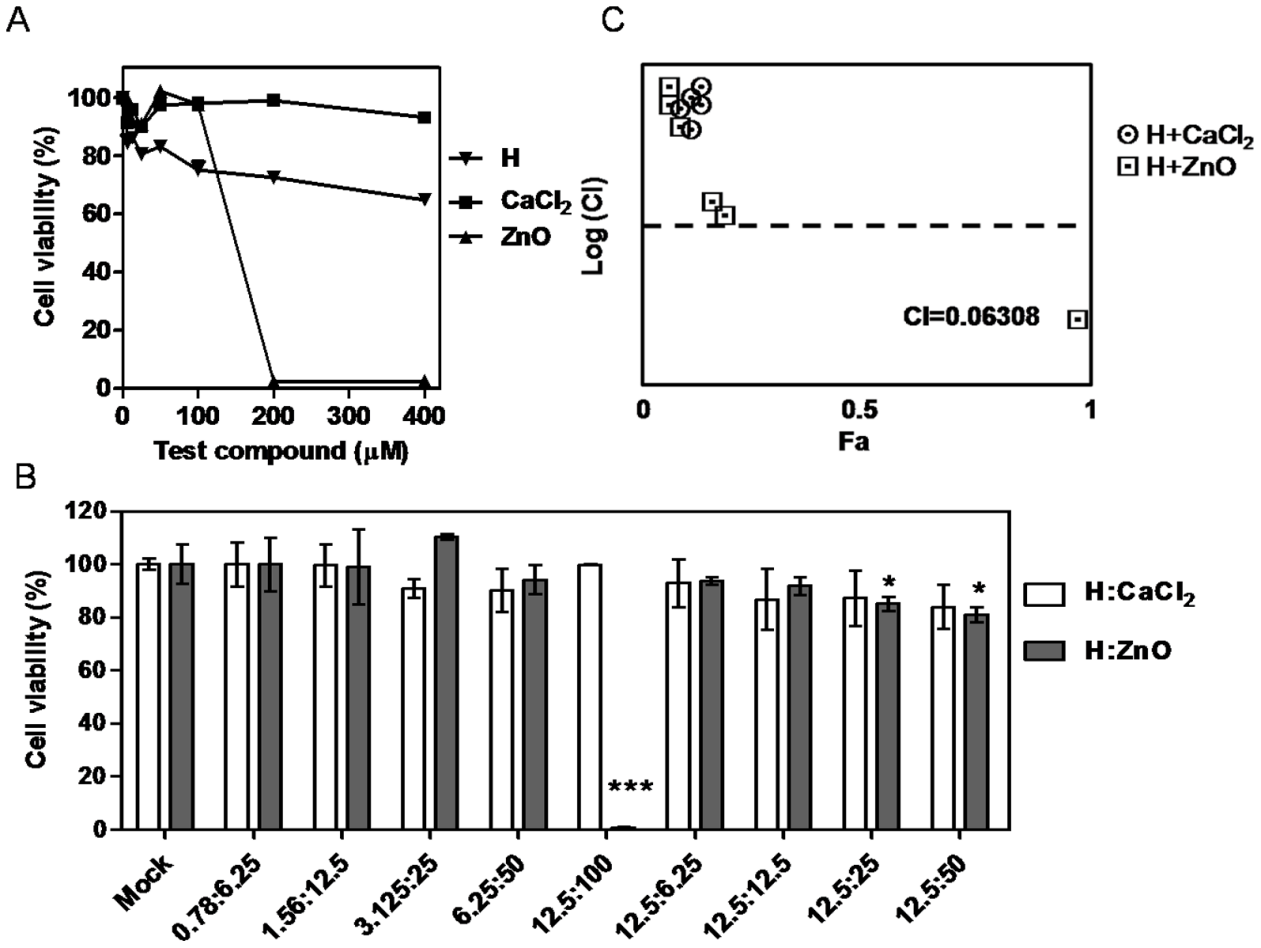
Combined hinokitiol and ZnO treatment created strong synergism. (A) MTT test analysis of the cytotoxicity of hinokitiol (H), CaCl_2_, and ZnO treated to MG-63 for 24 h. Vehicle (0.1% DMSO) and test compounds (6.25 µM-400 µM) were tested. Three independent experiments were performed in triplicate. (B) MTT test analysis of the cytotoxicity of combined various dosages of H:CaCl_2_, and H:ZnO to MG-63. Column, mean of more than triplicate analysis; bars, SE. (C) Logarithmic combination index plot for combo of H+CaCl_2_, and H+ZnO. Unpaired *t* test. *, *P*<0.05; ***, *P*<0.001.

CA+0.2%H exhibited a nonsignificantly different setting time and solubility but higher cytotoxicity, compared with MG-63. Unusually, the cytotoxicity disappeared entirely after 2 fold dilution (Fig. 2). The interaction between hinokitiol, a metal chelator, and Zn^2+^ in CA and Ca^2+^ in AP might have affected the sealer settings. It has been demonstrated that a combination of hinokitiol and Zn^2+^ enhanced the anti-*staphylococci* activity. Whether the hinokitiol, combined with Zn^2+^ in CA but not with Ca^2+^ in AP, caused the high cytotoxicity of MG-63 in CA+0.2%H is unclear. First, no significant cytotoxicity was observed in 100 µM CaCl_2_ and 100 µM ZnO, and the cell viability in 12.5 µM hinokitiol was 86% (Fig. 4A). Various combined dosage of hinokitiol:ZnO or hinokitiol:CaCl_2_ were tested, including a constant ratio (molar ratio: 1∶8, 0.78–12.5 µM:6.25–100 µM) and a nonconstant ratio (12.5 µM:6.25–100 µM). Finally, the cytotoxicity was enhanced when 12.5 µM hinokitiol was combined with 100 µM ZnO treatment, but not with CaCl_2_ (Fig. 4B). Synergism and additive and antagonistic effects were analyzed after the combined hinokitiol and ZnO treatment, using the Chou and Talalay combination index theorem. The logarithmic combination index plot in Fig. 4C (CI = 0.06308) shows that the combination of 12.5 µM hinokitiol and 100 µM ZnO treatment resulted in strong synergism.

### Physical characteristics of AP+0.5%–2%H conformed to ISO 6876:2001

Mixing 0.2% hinokitiol with the 3 sealers did not influence their physical characteristics. However, the original AH and CA already exhibited strong anti-MRSA abilities. Therefore, AH and CA were excluded from further study. AP did not exhibit anti-MRSA activity, but the solubility increased slightly after it was mixed with 0.2% hinokitiol. The physical characteristics, cytotoxicity, and anti-MRSA activity were not significantly different; therefore, AP tolerated more than 0.2% hinokitiol. An increased hinokitiol ratio in AP (0.5%H, 1%H, and 2%H) might improve its antibacterial activity. The setting time, film thickness, and solubility were dependent on the hinokitiol dose (0% to 2%) and increased, but the working time and flowability decreased. All of the test specimens conformed to ISO 6876:2001, except for the flowability of AP+2%. The detailed physical characteristics of AP with 0.5% to 2% hinokitiol are listed in Table 3.

**Table 3:** Physical tests of the AP contained 0.5%–2% hinokitiol (Mean (SD)).

	Setting time (h)	Working time (h)	Flowability (mm)	Film thickness (µm)	Solubility (%)
ISO 6876:2001	-	-	>20 mm	<50 µm	<3%
AP+0.5%H	5.08 (0.38)*	1.69 (0.1049)***	25.39 (1.01)***	14.00 (1.55)	1.62 (0.39)
AP+1%H	5.33 (0.29)**	1.08 (0.2357)***	25.72 (1.73)***	17.83 (1.60)***	2.03 (3.31)*
AP+2%H	6.17 (0.29)***	0.75 (0.22)***	18.34 (3.61)***	22.17 (2.32)***	2.30 (0.18)**

Entries are mean values with standard deviations in parentheses, and sample size (Mean (SD)). An unpaired *t* test was used to determine significant differences of the physical characteristics among the sealers contained various hinokitiol dosages in each group. The sample size of each group were 3 to 8. AP was control (Table 2). The asterisk indicates *P*<0.05, the double asterisk indicates *P*<0.01, and the triple asterisk indicates *P*<0.001.

### Cytotoxicity of AP+0.5%–2%H increased in a hinokitiol-dose-dependent manner

**Figure 5:**
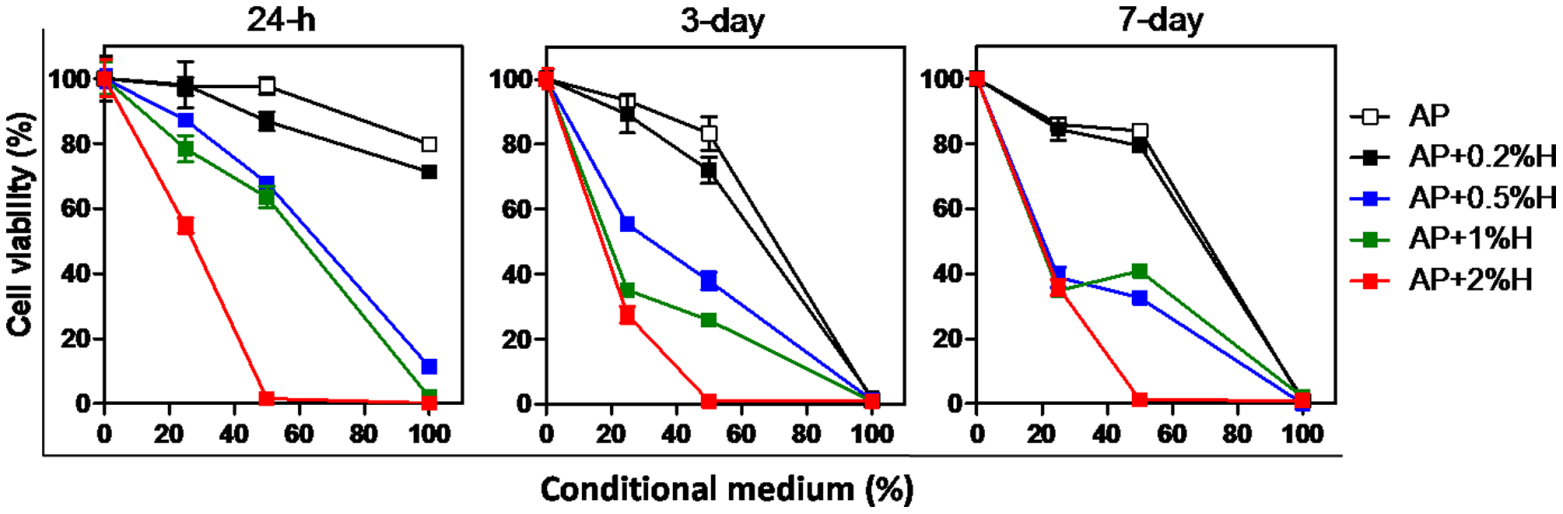
Cytotoxicity of Apexit Plus containing 0.2%–2% hinokitiol were increased by time and dose. (A) MTT test analysis of the cytotoxicity of extraction of the sealers (AP and AP+0.2%–2%H) conditional medium treated to MG-63 for 24 h (left panel), 3-days (middle panel), and 7-days (right panel). Two folds serial dilution of these conditional media (0%, 25%, 50%, and 100%) were tested. Three independent experiments were performed in triplicate.

The MG-63 viability was dose-dependent and decreased in relation to the hinokitiol percentage mixed with AP. The MG-63 viabilities of AP+0.5%H, 1%H, and 2%H conditional medium treatment for 24 h were approximately 10%, 2%, and 0%, respectively. The MG-63 viability of AP and AP+0.2%–2%H were completely inhibited when 100% conditional media were incubated for 3 and 7 d. The cytotoxicity of AP+0.5%–2%H increased in an incubation-time-dependent manner. After 25% and 50% conditional medium of AP+0.2%–2%H was treated for 1, 3 and 7 d, the cytotoxicity was reduced and countervailed by cell proliferation (Fig. 5).

### Antibacterial activity of AP+0.5%–2%H increased

**Figure 6:**
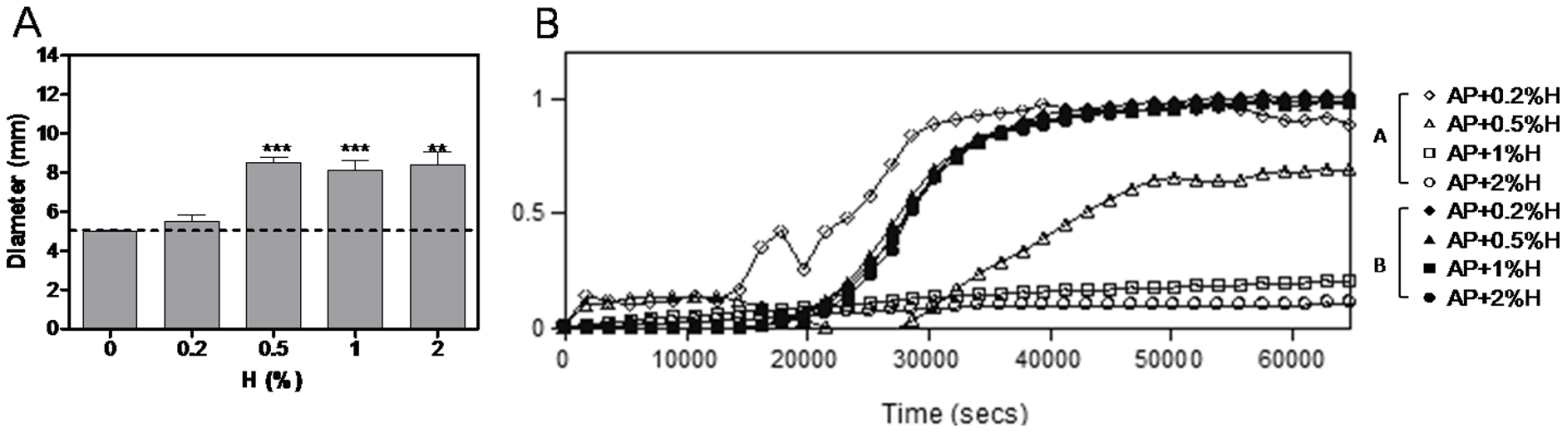
Anti-MRSA activity of Apexit Plus containing 0.2%–2% hinokitiol were increased. (A) Agar diffusion test analysis of anti-MRSA activity of the sealers (AP, AP+0.2%–2%H). Dot line, the diameter of the specimen (5 mm); column, mean of triplicate analysis; bars, SE. (C) Direct contact test analysis of the effect of close contact between test bacteria and the sealers on the kinetics of bacterial outgrowth. A was Group A (with AP+0.2%–2%H in the wells), and B was Group B (transfer 15 µl TSB from Group A and without sealers in the wells). Unpaired *t* test. **, *P*<0.01; ***, *P*<0.001.

In agar diffusion test, the anti-MRSA activity increased significantly in AP+0.5%H (8.50±0.29 mm, *P*<0.0001). The inhibition zone diameter remained the same in AP+1%H (8.13±0.52 mm) and AP+2%H (8.40±0.68 mm) (Fig. 6A). AP+0.5%H, 1%H, and 2%H exhibited similar inhibition diameters, suggesting that hinokitiol released a stable concentrate in the TSB agar plate. The direct contact test revealed that AP anti-MRSA activity depended on the dose of hinokitiol in Group A, but it did not differ significantly in Group B. The results were suggested that the AP mixed with hinokitiol did not eliminate MRSA after a 1-h exposure. The anti-MRAS activity of hinokitiol released from AP+0.5%–2%H continued for 18-h (Fig. 6B).

### Anti-inflammation potential of AP+0.5%–1%H increased

**Figure 7:**
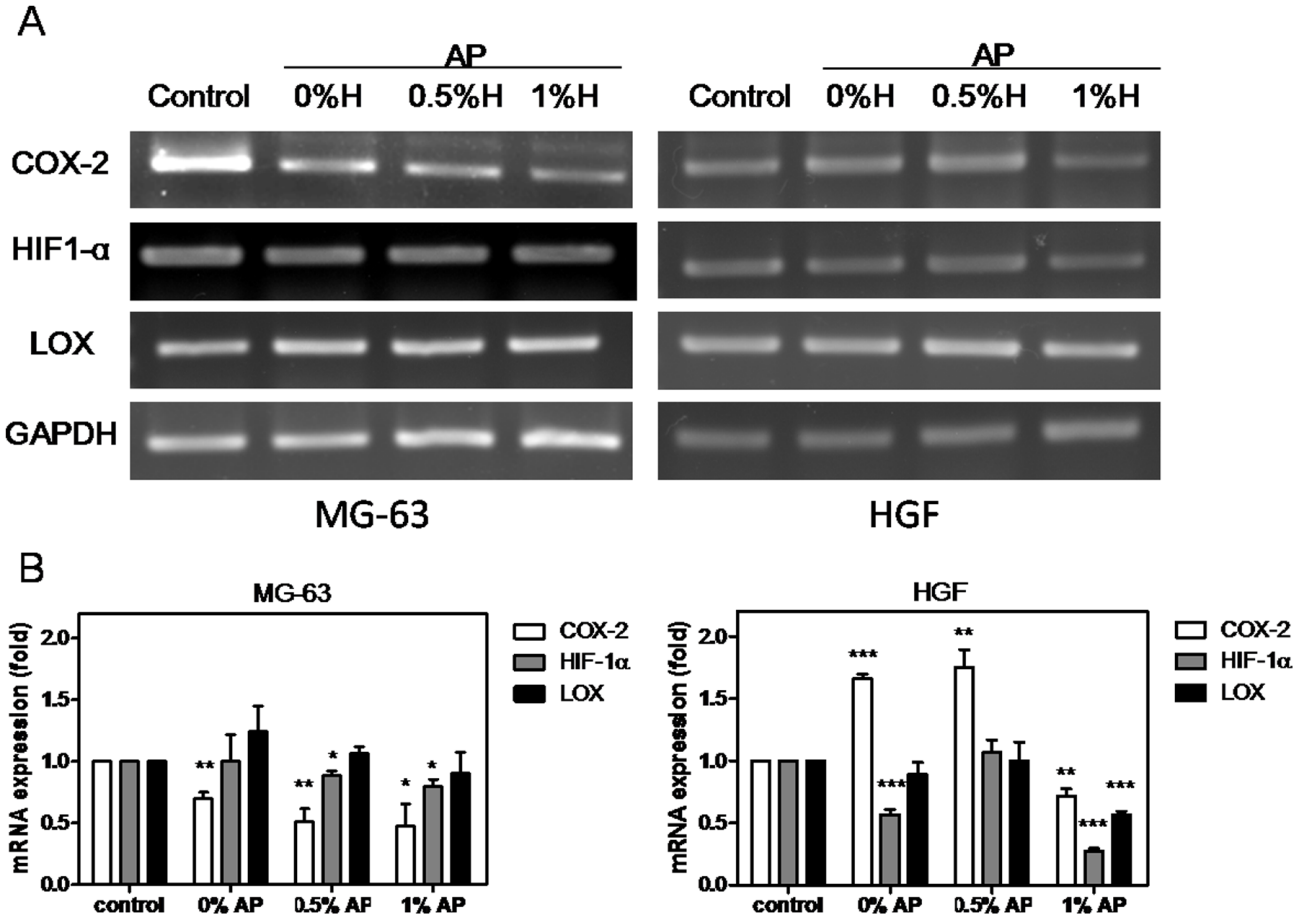
Anti-inflammation and anti-*S. aureus* abscess formation potential of Apexit Plus containing 0.5%–1% hinokitiol were increased. (A) The COX-2, HIF-1α, and LOX mRNA expression in MG-63 and HGF after AP, AP+0.5%H, and AP+1%H conditional medium incubated for 24 h. A normal cell culture conditional medium was used for the control. The COX-2, HIF-1α, LOX, and GAPDH expressions of MG-63 are shown in the left panel and of HGF are shown in the right panel. (B) Semi-quantitative the genes mRNA expression ratio to control in MG-63 and HGF cells. Three independent experiments were performed in triplicate. Column, mean of triplicate analysis; bars, SE. Unpaired *t* test. *, *P*<0.05; **, *P*<0.01; ***, *P*<0.001.

The anti-inflammation potentials of AP+0.5%H and AP+1%H were analyzed using RT-PCR to detect COX-2, HIF-1α, and LOX mRNA expressions in MG-63 and HGF. The AP+0%H conditional medium treatment induced COX-2 in HGF. The COX-2 expression was down-regulated by the AP+0.5%–1%H and AP+1%H conditional medium in MG-63 and HGF, respectively. The HIF-1*α* expression was down-regulated in MG-63 and HGF. The LOX expression did not differ significantly in MG-63 after AP+0.5%–1%H conditional medium incubation, but was inhibited in HGF after AP+1%H conditional medium incubation (Fig. 7A and 7B), indicating that AP induced cell inflammation but was inhibited by hinokitiol. The RT-PCR results suggested that AP with 0.5%–1% hinokitiol improved anti-inflammation and inhibited *S. aureus* abscess formation potentials.

## Discussion

The gram-positive bacteria, *Enterococcus faecalis*, MRSA, and *S. mutans*, and gram-negative bacteria, *A. actinomycetemcomitans*, were inhibited by hinokitiol. *E. faecalis*, *Staphylococcus spp.*, and *Streptococcus spp*. have been frequently observed in root canal-treated teeth. *S. aureus* and *S. mutans* were included in the microbial profiles of root filled teeth [32]. In a previous study, the paradoxical inhibition phenomenon (PIP) caused the first minimal inhibitory concentrations (MICs) of hinokitiol in *A. actinomycetemcomitans* and *S. mutans* to be 5 µM, and 10 µM, respectively [9]. The MIC of hinokitiol in *E. faecalis*, was 1.3 µg/mL (approximately 8 µM), but the PIP was not tested [33]. However, the PIP of penicillin was also determined in *E. faecalis* [34]. The MICs of *A. actinomycetemcomitans*, *S. mutans*, and *E. faecalis* were close to each other. MRSA was used in this study because of the high antimicrobial activity criteria of root canal sealers containing hinokitiol *in vitro*.

Previous studies have sequenced the antibacterial activity of the sealers as follows: zinc-oxide-eugenol-based > epoxy-resin-based > calcium-hydroxide-based root canal sealers [2]. Other studies have sequenced them as epoxy-resin-based > zinc-oxide-eugenol-based > calcium-hydroxide-based root canal sealers [3]. The various types of bacteria, commercial sealer products, and experimental conditions in each study explain the differences. However, the calcium-hydroxide-based root canal sealer has the lowest antibacterial activity in various studies, which is consistent with our data. The antibacterial activity of AH might release formaldehyde and ZrO_2_ [35], [36], AP might release hydroxyl ions and raise the pH value [37], and CA might release free eugenol and ZnO during the polymerization process [38]. AH exerted lower anti-MRSA effects than CA did in this study, possibly because only a small amount of formaldehyde was released [35]. AP exhibited strong antibacterial activity on gram-negative enterobacteria, such as *S. marcescens* and *E. coli.* [39], but showed less antibacterial activity on gram-positive *E. faecalis* [40], and MRSA than the other sealers did in this study. Soluble compounds were released from AP, inducing cytotoxicity (Fig. 2) but not anti-MRSA activity (Fig. 3A). The anti-MRSA activity of AP+0.2%H was from hinokitiol.

Hinokitiol exhibited a paradoxical inhibition phenomenon (PIP) regarding MRSA [5], [9], but this did not occur when the sealers were mixed with hinokitiol in this study. Hinokitiol, formaldehyde, ZrO_2_, eugenol, ZnO, and hydroxyl ions might have been released from the sealers. These compounds, in combination, resulted in each sealer having a unique inhibition zone diameter, and interfered with the hinokitiol PIP regarding MRSA [5]. We expected that the divalent metal Zn^2+^ might interact with hinokitiol to form Zn(hinokitiol)_2_ or other diketonates L to increase anti-MRSA activity [5], [41], [42]. However, the inhibition zone diameter of CA+0.2%H decreased. The inhibition zone diameter is associated with antibiotic diffusion effects, which include antibiotic concentration, molecular weight, solubility, and binding upon agar. Hinokitiol is a metal chelator. The setting times of AH, AP, and CA containing 0.2% hinokitiol all increased. Additional hinokitiol in CA might decrease release of free eugenol and ZnO, and induce new compound formation during setting. The cell viability of AH+0.2%H and AP+0.2%H decreased by approximately 10%, but CA+0.2%H decreased by more than 50% compared with the original sealers (Fig. 2). The solubility of AP+0.2%H increased significantly, but that of AH+0.2%H and CA+0.2%H did not (Table 2). Soluble compounds were released from AP, inducing cytotoxicity (Fig. 2), and the alkaline pH value of AP induced maximum cytotoxicity on the seventh day [43]. The cytotoxicity results of AH were consistent with Xu's study [27]. The CA+0.2%H revealed high cytotoxicity in this study because strong synergism is created when hinokitiol and Zn^2+^ combine, and the combined effect of hinokitiol and Ca^2+^ in AP can be excluded (Fig. 4).

The signs of failing root canal therapy are often discomfort and swelling, caused by intracanal bacteria and materials inducing inflammation. AP exhibited superior sealing ability and cellular compatibility [44], [45] (although more bacteria tended to adhere [46]), and lower antimicrobial activity in comparison with AH and Grossman's sealer [40], [47]. Both AP and Grossman's sealers initiated lymphocytic and plasmocytic reactions [48]. AP also induced inflammatory response [43], [49]. Additional 0.5% and 1% hinokitiol in AP significantly improved anti-MRSA activity, inhibited COX-2, HIF-1*α*, and LOX expression. The COX-2 and HIF-1*α* mediated inflammation differently. Additional hinokitiol in the calcium-hydroxide-based dental canal sealers might improve COX-2-associated inflammation, HIF-1α-associated inflammation, and LOX-associated *S. aureus* abscess formation potentials in vitro. The setting time, working time, flowability, film thickness, and solubility conformed to ISO 6876:2001, but increased cytotoxicity. However, the radiopacity, ball indentation hardness, hinokitiol release rate, and other safety tests must be conducted before future clinical application.

## Conclusions

AH and CA exhibited favorable anti-MRSA activity, but AP did not. AP+0.5%–1%H maintained stable physical characteristics, complying with ISO 6876:2001, and significantly improved anti-MRSA activity. Although the anti-MRSA activity of AP+0.5%–1%H was less than that of AH and CA, the anti-inflammation and the inhibited *S. aureus* abscess formation potential of AP+1%H were improved. ZnO is occasionally used to increase antibacterial activity in materials and the synergistic effect of hinokitiol and zinc combination can be applied in biomaterials or medical devices to increase their antibacterial activity, but also increase their cytotoxicity. These results indicated that an appreciable proportion of hinokitol is a beneficial compound to add to epoxy-amine-resin-based and calcium-hydroxide-based root canal sealers for improving the anti-MRSA activity and anti-inflammation potential, and inhibiting *S. aureus* abscess formation; however, the enhanced cytotoxicity and synergistic effect must be considered.

## Supporting Information

Table S1 **Commercial dental sealers and hinokitiol mixture percentage.**
(DOCX)

## References

[1] TRP F (1997) Harty's Endodontics in Clinical Practice.

[2] Saha S, Samadi F, Jaiswal JN, Ghoshal U (2010) Antimicrobial activity of different endodontic sealers: an in vitro evaluation. J Indian Soc Pedod Prev Dent 28: 251–257. 21273712 10.4103/0970-4388.76151

[3] Shantiaee Y, Dianat O, Janani A, Kolahi Ahari G (2010) In vitro evaluation of the antibacterial activity of three root canal sealers. Iran Endod J 5: 1–5. 23130021 PMC3471567

[4] Inamori Y, Sakagami Y, Morita Y, Shibata M, Sugiura M, et al (2000) Antifungal activity of Hinokitiol-related compounds on wood-rotting fungi and their insecticidal activities. Biol Pharm Bull 23: 995–997. 10963310 10.1248/bpb.23.995

[5] Arima Y, Nakai Y, Hayakawa R, Nishino T (2003) Antibacterial effect of beta-thujaplicin on staphylococci isolated from atopic dermatitis: relationship between changes in the number of viable bacterial cells and clinical improvement in an eczematous lesion of atopic dermatitis. J Antimicrob Chemother 51: 113–122. 12493795 10.1093/jac/dkg037

[6] Krenn BM, Gaudernak E, Holzer B, Lanke K, Van Kuppeveld FJ, et al (2009) Antiviral activity of the zinc ionophores pyrithione and hinokitiol against picornavirus infections. J Virol 83: 58–64. 18922875 10.1128/JVI.01543-08 PMC2612303

[7] Ema M, Harazono A, Fujii S, Kawashima K (2004) Evaluation of developmental toxicity of beta-thujaplicin (hinokitiol) following oral administration during organogenesis in rats. Food Chem Toxicol 42: 465–470. 14871589 10.1016/j.fct.2003.10.009

[8] Imai N, Doi Y, Nabae K, Tamano S, Hagiwara A, et al (2006) Lack of hinokitiol (beta-thujaplicin) carcinogenicity in F344/DuCrj rats. J Toxicol Sci 31: 357–370. 17077589 10.2131/jts.31.357

[9] Shih YH, Chang KW, Hsia SM, Yu CC, Fuh LJ, et al (2013) In vitro antimicrobial and anticancer potential of hinokitiol against oral pathogens and oral cancer cell lines. Microbiol Res 168: 254–262. 23312825 10.1016/j.micres.2012.12.007

[10] Saeki Y, Ito Y, Shibata M, Sato Y, Okuda K, et al (1989) Antimicrobial action of natural substances on oral bacteria. Bull Tokyo Dent Coll 30: 129–135. 2637783

[11] Nagao Y, Sata M (2011) Effect of oral care gel on the quality of life for oral lichen planus in patients with chronic HCV infection. Virol J 8: 348. 21749712 10.1186/1743-422X-8-348 PMC3149004

[12] Higashi Y, Sakata M, Fujii Y (2009) High-performance liquid chromatography with dual-wavelength ultraviolet detection for measurement of hinokitiol in personal care products. J Cosmet Sci 60: 519–525. 19822109

[13] Iha K, Suzuki N, Yoneda M, Takeshita T, Hirofuji T (2013) Effect of mouth cleaning with hinokitiol-containing gel on oral malodor: a randomized, open-label pilot study. Oral Surg Oral Med Oral Pathol Oral Radiol 10.1016/j.oooo.2013.05.021 23969334

[14] Lin KH, Kuo JR, Lu WJ, Chung CL, Chou DS, et al (2013) Hinokitiol inhibits platelet activation ex vivo and thrombus formation in vivo. Biochem Pharmacol 85: 1478–1485. 23473801 10.1016/j.bcp.2013.02.027

[15] Shih MF, Chen LY, Tsai PJ, Cherng JY (2012) In vitro and in vivo therapeutics of beta-thujaplicin on LPS-induced inflammation in macrophages and septic shock in mice. Int J Immunopathol Pharmacol 25: 39–48. 22507316 10.1177/039463201202500106

[16] Byeon SE, Lee YG, Kim JC, Han JG, Lee HY, et al (2008) Hinokitiol, a natural tropolone derivative, inhibits TNF-alpha production in LPS-activated macrophages via suppression of NF-kappaB. Planta Med 74: 828–833. 18537078 10.1055/s-2008-1074548

[17] Oh YT, Lee JY, Yoon H, Lee EH, Baik HH, et al (2008) Lipopolysaccharide induces hypoxia-inducible factor-1 alpha mRNA expression and activation via NADPH oxidase and Sp1-dependent pathway in BV2 murine microglial cells. Neurosci Lett 431: 155–160. 18164813 10.1016/j.neulet.2007.11.033

[18] Maxwell PH, Pugh CW, Ratcliffe PJ (2001) Activation of the HIF pathway in cancer. Curr Opin Genet Dev 11: 293–299. 11377966 10.1016/s0959-437x(00)00193-3

[19] Ng KT, Li JP, Ng KM, Tipoe GL, Leung WK, et al (2011) Expression of hypoxia-inducible factor-1alpha in human periodontal tissue. J Periodontol 82: 136–141. 21043802 10.1902/jop.2010.100100

[20] Hellwig-Burgel T, Stiehl DP, Wagner AE, Metzen E, Jelkmann W (2005) Review: hypoxia-inducible factor-1 (HIF-1): a novel transcription factor in immune reactions. J Interferon Cytokine Res 25: 297–310. 15957953 10.1089/jir.2005.25.297

[21] Huang FM, Chou MY, Chang YC (2003) Induction of cyclooxygenase-2 mRNA and protein expression by epoxy resin and zinc oxide-eugenol based root canal sealers in human osteoblastic cells. Biomaterials 24: 1869–1875. 12615477 10.1016/s0142-9612(02)00584-7

[22] Jung YJ, Isaacs JS, Lee S, Trepel J, Neckers L (2003) IL-1beta-mediated up-regulation of HIF-1alpha via an NFkappaB/COX-2 pathway identifies HIF-1 as a critical link between inflammation and oncogenesis. FASEB J 17: 2115–2117. 12958148 10.1096/fj.03-0329fje

[23] Mariani F, Sena P, Marzona L, Riccio M, Fano R, et al (2009) Cyclooxygenase-2 and Hypoxia-Inducible Factor-1alpha protein expression is related to inflammation, and up-regulated since the early steps of colorectal carcinogenesis. Cancer Lett 279: 221–229. 19268443 10.1016/j.canlet.2009.02.001

[24] Beerlage C, Greb J, Kretschmer D, Assaggaf M, Trackman PC, et al (2013) Hypoxia-inducible factor 1-regulated lysyl oxidase is involved in Staphylococcus aureus abscess formation. Infect Immun 81: 2562–2573. 23649089 10.1128/IAI.00302-13 PMC3697610

[25] Lu H, Ouyang W, Huang C (2006) Inflammation, a key event in cancer development. Mol Cancer Res 4: 221–233. 16603636 10.1158/1541-7786.MCR-05-0261

[26] Chang PY, Peng SF, Lee CY, Lu CC, Tsai SC, et al (2013) Curcumin-loaded nanoparticles induce apoptotic cell death through regulation of the function of MDR1 and reactive oxygen species in cisplatin-resistant CAR human oral cancer cells. Int J Oncol 43: 1141–1150. 23917396 10.3892/ijo.2013.2050

[27] Xu P, Liang J, Dong G, Zheng L, Ye L (2010) Cytotoxicity of RealSeal on human osteoblast-like MG63 cells. J Endod 36: 40–44. 20003933 10.1016/j.joen.2009.09.002

[28] Shih YH, Chang KW, Chen MY, Yu CC, Lin DJ, et al (2013) Lysyl oxidase and enhancement of cell proliferation and angiogenesis in oral squamous cell carcinoma. Head Neck 35: 250–256. 22367676 10.1002/hed.22959

[29] Weiss EI, Shalhav M, Fuss Z (1996) Assessment of antibacterial activity of endodontic sealers by a direct contact test. Endod Dent Traumatol 12: 179–184. 9028181 10.1111/j.1600-9657.1996.tb00511.x

[30] Anumula L, Kumar S, Kumar VS, Sekhar C, Krishna M, et al (2012) An Assessment of Antibacterial Activity of Four Endodontic Sealers on Enterococcus faecalis by a Direct Contact Test: An In Vitro Study. ISRN Dent 2012: 989781. 22888444 10.5402/2012/989781 PMC3408652

[31] Shieh TM, Chang KW, Tu HF, Shih YH, Ko SY, et al (2010) Association between the polymorphisms in exon 12 of hypoxia-inducible factor-1alpha and the clinicopathological features of oral squamous cell carcinoma. Oral Oncol 46: e47–53. 20656543 10.1016/j.oraloncology.2010.04.009

[32] Anderson AC, Hellwig E, Vespermann R, Wittmer A, Schmid M, et al (2012) Comprehensive analysis of secondary dental root canal infections: a combination of culture and culture-independent approaches reveals new insights. PLoS One 7: e49576. 23152922 10.1371/journal.pone.0049576 PMC3495864

[33] Oblak EZ, Bolstad ES, Ononye SN, Priestley ND, Hadden MK, et al (2012) The furan route to tropolones: probing the antiproliferative effects of beta-thujaplicin analogs. Org Biomol Chem 10: 8597–8604. 23032214 10.1039/c2ob26553b

[34] Fontana R, Boaretti M, Grossato A, Tonin EA, Lleo MM, et al (1990) Paradoxical response of Enterococcus faecalis to the bactericidal activity of penicillin is associated with reduced activity of one autolysin. Antimicrob Agents Chemother 34: 314–320. 2109578 10.1128/aac.34.2.314 PMC171579

[35] Leonardo MR, Bezerra da Silva LA, Filho MT, Santana da Silva R (1999) Release of formaldehyde by 4 endodontic sealers. Oral Surg Oral Med Oral Pathol Oral Radiol Endod 88: 221–225. 10468467 10.1016/s1079-2104(99)70119-8

[36] Jangra SL, Stalin K, Dilbaghi N, Kumar S, Tawale J, et al (2012) Antimicrobial activity of zirconia (ZrO2) nanoparticles and zirconium complexes. J Nanosci Nanotechnol 12: 7105–7112. 23035440 10.1166/jnn.2012.6574

[37] Cvek M (1974) Treatment of non-vital permanent incisors with calcium hydroxide. IV. Periodontal healing and closure of the root canal in the coronal fragment of teeth with intra-alveolar fracture and vital apical fragment. A follow-up. Odontol Revy 25: 239–246. 4530952

[38] Hume WR (1986) The pharmacologic and toxicological properties of zinc oxide-eugenol. J Am Dent Assoc 113: 789–791. 3537057 10.14219/jada.archive.1986.0256

[39] Sonja Pezelj-Ribarić IB, Miletić Ivana, Abram Maja, Karlović Zoran, Anić Ivica (2001) Antibacterial Activity of Calcium Hydroxide Root Canal Sealer (Apexit) - in vitro Study. Acta Stomatol Croat 35: 475–477.

[40] Kayaoglu G, Erten H, Alacam T, Orstavik D (2005) Short-term antibacterial activity of root canal sealers towards Enterococcus faecalis. Int Endod J 38: 483–488. 15946270 10.1111/j.1365-2591.2005.00981.x

[41] Liguori PF, Valentini A, Palma M, Bellusci A, Bernardini S, et al (2010) Non-classical anticancer agents: synthesis and biological evaluation of zinc(II) heteroleptic complexes. Dalton Trans 39: 4205–4212. 20390185 10.1039/b922101h

[42] Barret MC, Mahon MF, Molloy KC, Steed JW, Wright P (2001) Synthesis and structural characterization of tin(II) and zinc(II) derivatives of cyclic alpha-hydroxyketones, including the structures of Sn(maltol)(2), Sn(tropolone)(2), Zn(tropolone)(2), and Zn(hinokitiol)(2). Inorg Chem 40: 4384–4388. 11487346 10.1021/ic0100368

[43] Yesilsoy C, Koren LZ, Morse DR, Kobayashi C (1988) A comparative tissue toxicity evaluation of established and newer root canal sealers. Oral Surg Oral Med Oral Pathol 65: 459–467. 3163136 10.1016/0030-4220(88)90361-1

[44] Salz U, Poppe D, Sbicego S, Roulet JF (2009) Sealing properties of a new root canal sealer. Int Endod J 42: 1084–1089. 19912379 10.1111/j.1365-2591.2009.01635.x

[45] Schwarze T, Fiedler I, Leyhausen G, Geurtsen W (2002) The cellular compatibility of five endodontic sealers during the setting period. J Endod 28: 784–786. 12470025 10.1097/00004770-200211000-00009

[46] Senges C, Wrbas KT, Altenburger M, Follo M, Spitzmuller B, et al (2011) Bacterial and Candida albicans adhesion on different root canal filling materials and sealers. J Endod 37: 1247–1252. 21846541 10.1016/j.joen.2011.05.034

[47] Saleh IM, Ruyter IE, Haapasalo M, Orstavik D (2004) Survival of Enterococcus faecalis in infected dentinal tubules after root canal filling with different root canal sealers in vitro. Int Endod J 37: 193–198. 15009409 10.1111/j.0143-2885.2004.00785.x

[48] Bernath M, Szabo J (2003) Tissue reaction initiated by different sealers. Int Endod J 36: 256–261. 12702119 10.1046/j.1365-2591.2003.00662.x

[49] Silva LA, Leonardo MR, Faccioli LH, Figueiredo F (1997) Inflammatory response to calcium hydroxide based root canal sealers. J Endod 23: 86–90. 9220736 10.1016/s0099-2399(97)80251-8

