# Supplementary material for: Evaluation Physical Characteristics and Comparison Antimicrobial and Anti-Inflammation Potentials of Dental Root Canal Sealers Containing Hinokitiol In Vitro
Source: PLoS One. 2014 Jun 10;9(6):e94941. doi: 10.1371/journal.pone.0094941 (PMC4051635; doi:10.1371/journal.pone.0094941)
Supplement: Table S1 — Commercial dental sealers and hinokitiol mixture percentage. (DOCX) [file pone.0094941.s001.docx]

**Supporting Information**

Table S1.Commercial dental sealers and hinokitiol mixture percentage

| Specimen | Paste A/Paste B^a^ or Powder/Liquid^b^ (g) | Hinokitiol (g) |
| --- | --- | --- |
| AH Plus (AH) | ^a^ 0.57 / 0.57 | 0 |
| AH Plus+0.2% Hinokitiol (AH+0.2%H) | ^a^ 0.57 / 0.57 | 0.003 |
| Apexit Plus (AP) | ^a^ 0.57 / 0.56 | 0 |
| Apexit Plus+0.2% Hinokitiol (AP+0.2%H) | ^a^ 0.57 / 0.56 | 0.003 |
| Apexit Plus+0.5% Hinokitiol (AP+0.5%H) | ^a^ 0.57 / 0.57 | 0.0075 |
| Apexit Plus+1% Hinokitiol (AP+1%H) | ^a^ 0.57 / 0.57 | 0.015 |
| Apexit Plus+2% Hinokitiol (AP+2%H) | ^a^ 0.57 / 0.57 | 0.03 |
| Canals (CA) | ^b^ 0.93 / 0.15 | 0 |
| Canals+2% Hinokitiol (CA+0.2%H) | ^b^ 0.93 / 0.15 | 0.002 |

1. The sealer components are paste A + paste B
2. The sealer components are powder + liquid
